# Tunnelling Nanotube Projections May Interfere with *Toxoplasma gondii* Interaction with Host Cells

**DOI:** 10.3390/microorganisms14050971

**Published:** 2026-04-26

**Authors:** Everson Reili de Souza Teles, Wanderley de Souza

**Affiliations:** 1 Laboratório de Ultraestrutura Celular Hertha Meyer, Centro de Pesquisa em Medicina de Precisão, Instituto de Biofísica Carlos Chagas Filho, Centro Nacional de Biologia Estrutural e Bioimagem, Universidade Federal do Rio de Janeiro, Rio de Janeiro 21941-900, Brazil; everson.rsteles@gmail.com; 2 Centro Multiusuário de Pesquisa em Fenômenos Biomédicos (CMABIO), Escola Superior de Saúde, Universidade do Estado do Amazonas, Manaus 69065-001, Brazil

**Keywords:** *Toxoplasma gondii*, parasite-host cell adhesion and interaction, fluorescence microscopy, electron microscopy, tunneling nanotubes

## Abstract

*Toxoplasma gondii*, the causative agent of toxoplasmosis, a disease widely distributed, is an intracellular parasite that invades host cells of different tissues using specialized endocytic activity. Recent studies suggest that tunneling nanotubes (TNTs), thin cell-surface projections, may participate in the parasite–host cell interaction. Here we report results that suggest the involvement of host-cell TNTs in the adhesion of *T. gondii* tachyzoites to epithelial LLC-MK^2^ cells. Microscopy analysis showed that incubating cells in a medium containing 0.45 M sucrose induces reversible assembly of TNTs without affecting cell viability. The presence of extended TNTs correlated with increased parasite adhesion and reduced parasite entry, thus suggesting a structural or signaling role in mediating adhesion. TNTs assembled following sucrose incubation contain both actin and tubulin components as determined by immunofluorescence microscopy. These results highlight a possible functional relevance of TNTs in *T. gondii* host cell interaction, especially in parasite adhesion, opening new perspectives for understanding *T. gondii*-host cell interaction.

## 1. Introduction

*Toxoplasma gondii* is an intracellular parasitic protist belonging to the phylum Apicomplexa. It is the etiological agent of human and animal toxoplasmosis, widely distributed worldwide, that can infect nearly all warm-blooded animals, including humans. It is estimated that approximately 30% of the global human population is infected with this parasite. It actively invades host cells, using highly specialized mechanisms known as induced endocytosis, involving parasite motility, adhesion to the surface of the host cell, extrusion of the conoid, and release of proteins existing in the micronemes and rhoptries of the parasite, culminating in the formation of a parasitophorous vacuole located within the host cell [1,2,3,4]. There are few studies about the changes that occur on the surface of the host cell during the interaction process. Using high-resolution scanning electron microscopy, we showed previously that occasionally we observed the presence of structures known as Tunneling Nanotubes (TNTs), which emerge from the surface of the host cell and establish contact with the parasite itself, even at long distances from the host cell surface [5], suggesting a possible participation of TNTs in the *T. gondii*-host cell interaction process. TNTs are plasma membrane protrusions containing actin filaments and microtubules, displaying variable diameters and reaching distances as long as 400 μm. They establish contact with another cell, forming a kind of nanotube that allows cell-to-cell communication and the transfer of macromolecular complexes, and even organelles, between them. TNTs were initially described by Kornberg (1999) in *Drosophila* [6] and later found in many cell types [7], including immune cells [8]. The thin, elongated extensions of the plasma membrane are based on actin and, depending on the cell type, tubulin. TNTs differ from other cellular structures by their lack of contact with the substrate and their direct connection to the cell cytoplasm [9]. It is an additional way to connect cells, extremely relevant to intercellular signaling. It allows direct cell-to-cell transfer of biological materials. Our previous initial morphological information showed a close association of *T. gondii* tachyzoites with TNTs. The present study aims to analyze further the interaction of *T. gondii* tachyzoites with cells that have been stimulated to assemble many TNTs. Our results show that during host cell-parasite interaction, there is a clear association of the parasite with TNTs that increases parasite adhesion but decreases parasite invasion of host cells.

## 2. Materials and Methods

### 2.1. Host Cells

The host cell lineage used in the experiments was the epithelial cells LLC-MK^2^ (ATCC-CCL7, Rockville, MD, USA) from the monkey kidney Rhesus (*Macaca mullata*), grown in RPMI (Roswell Park Memorial Institute) 1640 medium (Thermo Fisher Scientific, Waltham, MA, USA) supplemented with 10% heat-inactivated bovine fetal serum (FBS; GIBCO, Thermo Fisher Scientific, Waltham, MA, USA). The cell lineage was grown on glass coverslips or in culture flasks and maintained at 37 °C in a 5% CO_2_ atmosphere.

### 2.2. Parasite Maintenance

*T. gondii* tachyzoites (RH strain) were maintained by passages in LLC-MK^2^ cells at confluence. After 1 day of infection, parasites were obtained from the supernatant, centrifuged at 1000× *g* for 10 min, and resuspended in 10 mL of RPMI medium. The number of parasites was quantified using a Neubauer chamber (Marienfeld, Lauda-Königshofen, Germany).

### 2.3. Interaction Assay

One day before the interaction, 5 × 10^5^ cells were seeded on coverslips placed in a 24-well plate or directly on a 96-well plate. Then, the cells were washed 3 times with phosphate-buffered saline (PBS), pH 7.2. Subsequently, the samples were incubated at 37 °C in a 5% CO_2_ atmosphere. The parasites were added at a 50:1 ratio (parasites per host cell) for 30 min. After interaction with the parasites, the cells were washed three times with PBS to remove non-adherent parasites and confirm persistent adhesion. The samples were fixed in a solution containing 3% freshly prepared formaldehyde and 0.1% glutaraldehyde in PBS for 15 min, or in 2.5% glutaraldehyde in 0.1 M cacodylate buffer, pH 7.2, for light and electron microscopy, respectively. To determine adhesion and internalization indices after the interaction, the cultures were fixed in a 4% formaldehyde solution in PBS (pH 7.2) and stained with 10% Giemsa. The samples were dehydrated in different concentrations of acetone-xylol. The coverslips were mounted on Entellan^®^ (Merck Millipore, Darmstadt, Germany) drops and analyzed under a light microscope using an Axion Zeiss microscope (Axio Imager, Zeiss, Oberkochen, Germany) with a 100× oil-immersion objective. To distinguish adhered from internalized parasites, we carefully examined many samples and used the presence of a parasitophorous vacuole as a criterion for identifying internalized parasites. The adhered parasites were easily identified in the samples by a simple change in the focus plane since they are located on the host cell surface. The adhesion index was obtained by multiplying the mean number of parasites adhered to the host cell and the percentage of cells with adhered parasites. The internalization index was calculated by multiplying the mean number of internalized parasites per infected cell and the percentage of infected cells [10]. The data were obtained after analysis of three different coverslips per experiment. The data were plotted using GraphPad Prism 9.0. The presented results represent the means ± standard deviations from at least three independent experiments, and differences were considered statistically significant at *p* < 0.05.

In order to determine the association of tachyzoites with induced TNTs, we analyzed a total of 150 SEM images of sucrose treated cells.

### 2.4. Induction of Tunneling Nanotube Assembly

Nanotubes were induced by incubating the cells with 0.45 M sucrose for 30 min at 37 °C, washing with PBS (pH 7.4), and then supplementing with RPMI medium [11]. These parameters were established based on previous studies conducted in our laboratory to analyze the process of parasitic protozoa–host cell interaction [11]. They were allowed to interact with the parasites, as described above. To distinguish the effects of sucrose treatment from TNT formation, all assays included appropriate controls without sucrose exposure. It is important to point out that the sucrose-treated cells are viable, and that the effect was reversible, as replacing the sucrose medium with the culture medium drastically reduced the number of TNTs. TNTs were always identified based on localization of actin filaments using fluorescence microscopy, as described below.

### 2.5. Cell Viability

To assess the effect of sucrose treatment and the reversibility of the TNT induction process, LLC-MK^2^ cells were seeded at 2.5 × 10^5^ cells/well in a 96-well plate. They were incubated in RPMI supplemented with 10% FCS. After 24 h of growth, they were incubated for 1 h in 0.45 M of sucrose in RPMI. To assess cell viability after incubation, they were washed with PBS (pH 7.2), incubated in RPMI supplemented with 10% FCS for 20 min, and cell viability was then assessed using the MTS/PMS assay. Quantification was made by measuring optical density at 490 nm using a BioTek Synergy H1 spectrofluorometer (BioTek Instruments, Winooski, VT, USA). Three independent experiments were performed in triplicate. The values were calculated by fitting them to a percentage viability analysis after reversal of the sucrose treatment. Regression analyses were performed using SigmaPlot 10.

### 2.6. Fluorescence Microscopy

For the analysis of actin presence in tunneling nanotubes (TNTs), we used the classical fluorescence microscopy approach to label actin filaments. The cells were fixed in a solution containing 4% formaldehyde in PBS (pH 7.2) for 20 min, washed with PBS (pH 7.2), and permeabilized in a solution containing 0.05% saponin and 3% bovine serum albumin (BSA) in PBS for 10 min. Subsequently, the samples were sequentially incubated in a blocking solution (0.05% saponin and 3% BSA in PBS) for 30 min at room temperature. The cells were then incubated with mouse anti-tubulin polyclonal antibody (1:2000 dilution) (Sigma-Aldrich, St. Louis, MO, USA) for 1 h. Next, the cells were washed with the blocking solution and incubated with Alexa Fluor 488 anti-rabbit secondary antibody (1:800 dilution) (Molecular Probes, Thermo Fisher Scientific, Eugene, OR, USA) for 1 h, protected from light. The samples were then incubated for 1 h with fluorescent phalloidin 546 (1:40 dilution) (Molecular Probes, Thermo Fisher Scientific, Eugene, OR, USA), a fungal peptide that binds to actin filaments. Observations were performed using the ZEISS LSM88O confocal microscope (Carl Zeiss AG, Jena, Germany) at a magnification of 63×.

### 2.7. Scanning Electron Microscopy

For morphological analysis of the initial events of the interaction by scanning electron microscopy, the interaction assay was performed, as described above. After 15 min of interaction, the samples were washed once with PBS (pH 7.5) and fixed with a solution containing 2.5% glutaraldehyde in 0.1 M sodium cacodylate buffer (pH 7.2). Then, the cells were washed in PBS, postfixed in 1% OsO_4_ (Osmium Tetroxide), and dehydrated in ethanol (30–100%) before being critically point dried with liquid CO_2_. The samples were coated with a 5 nm thick platinum layer and observed in a Zeiss Auriga 40 scanning electron microscope (Carl Zeiss AG, Oberkochen, Germany) operating at 4 kV. For Ion microscopy observation, the samples were not coated and were observed in an ORION model Helium Ion Scanning microscope (HIM) (Zeiss Nanofab, Peabody, MA, USA) with tungsten filament and operated at 35 kV, with a working distance of 8.7 mm, pressure of 5.5 × 10^−6^ Torr, and beam current of 0.8 pA.

### 2.8. Transmission Electron Microscopy

Parasite–host cell interaction was carried out as described above. The cells were fixed in a solution containing 2.5% cacodylate buffer, pH 7.2, post-fixed in 1% osmium tetroxide, dehydrated in ethanol-glutaraldehyde in 0.1 M cacodylate buffer, pH 7.2, post-fixed in 1% osmium tetroxide, dehydrated in ethanol, and embedded in Epoxi Resin. Thin sections were stained with uranyl acetate and lead citrate and observed in a transmission electron microscope (Hitachi or FEI, Hitachi High-Technologies Corp., Tokyo, Japan) operating at 100 kV.

## 3. Results

### 3.1. Morphological Observations

In view of our previous observation of host cells’ TNTs interacting with *T. gondii*, we decided to use an experimental system where the assembly of such structures is stimulated by incubation in the presence of 0.45 M sucrose [12] as shown in our previous studies [11,13]. After pretreatment of LLC-MK^2^ cells with 0.45 M sucrose for 1 h, followed by washing and incubation in medium supplemented with 10% fetal bovine serum, the cytotoxic effect of sucrose on the cells was evaluated. After removal of sucrose from the medium, the TNTs decreased both in number and length.

We performed a fluorescence microscopy assay to evaluate the presence of actin in tunneling nanotubes (TNTs). The samples were stained to identify actin filaments, using Phalloidin, and microtubules, using anti-tubulin antibodies, enabling visualization of cytoskeletal organization along these structures. The acquired images revealed a continuous fluorescent signal along the TNTs, consistent with the presence of actin filaments, supporting their role in the structural composition of these cellular protrusions. These findings confirm that the TNTs, as observed by scanning electron microscopy, exhibit an actin-based framework, consistent with classical descriptions in the literature [9].

**Figure 1:**
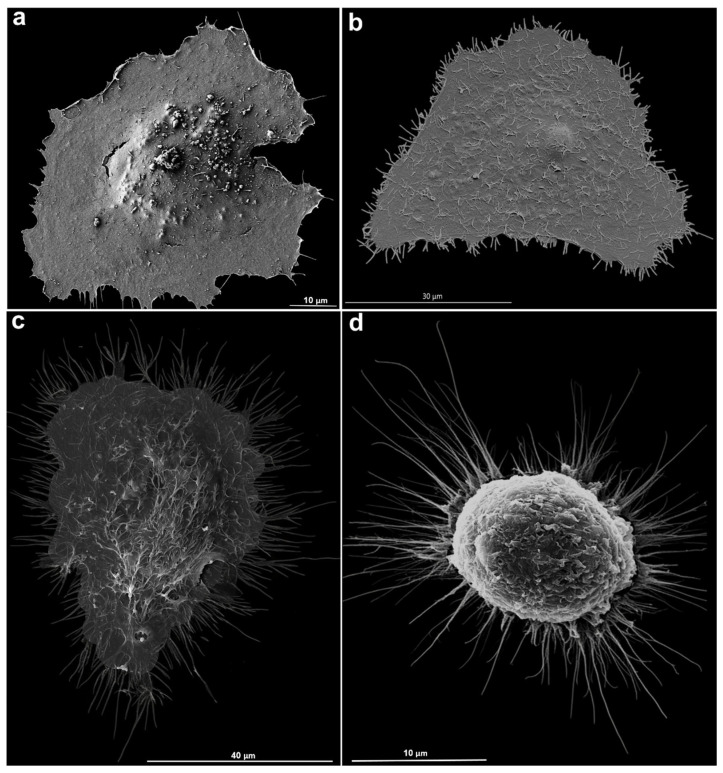
Morphological aspects of the surface of the LLC MK^2^ cell line observed by scanning electron microscopy. (**a**). Classical epithelial organization, with elongated/polygonal cells and well-defined membrane projections. (**b**). Short TNTs, with lengths between 900 nm and 5 µm. (**c**). Cells treated with sucrose: TNTs longer than 5 µm. (**d**). Rounding of the cellular body, and the presence of TNTs up to 20 µm long.

Using scanning electron microscopy, we observed that LLC MK2 cells exhibited a well-preserved morphology, with a slight rounding, compared to untreated cells (Figure 1a,b). It is important to point out that the cell viability assay indicated that the sucrose treatment did not affect the cells and that the induction of the TNT assembly was reversed after transference of the cells to the RPMI medium. The results presented in Figure 1 reveal significant morphological changes on the surface of LLC-MK^2^ cells induced by sucrose treatment, as observed by scanning electron microscopy. In Figure 1a, a classical epithelial organization is evident, with elongated or polygonal cells and with few surface protrusions, representing the basic morphology of untreated cells. Figure 1b highlights the presence of a large number of short TNTs, suggesting that these structures are present under physiological conditions, albeit in a limited number and length. However, sucrose treatment leads to notable morphological alterations: in Figure 1c, a clear increase in TNT length is observed, with structures exceeding 5 µm. This effect is further intensified, as shown in Figure 1d, where longer TNTs are observed.

**Figure 2:**
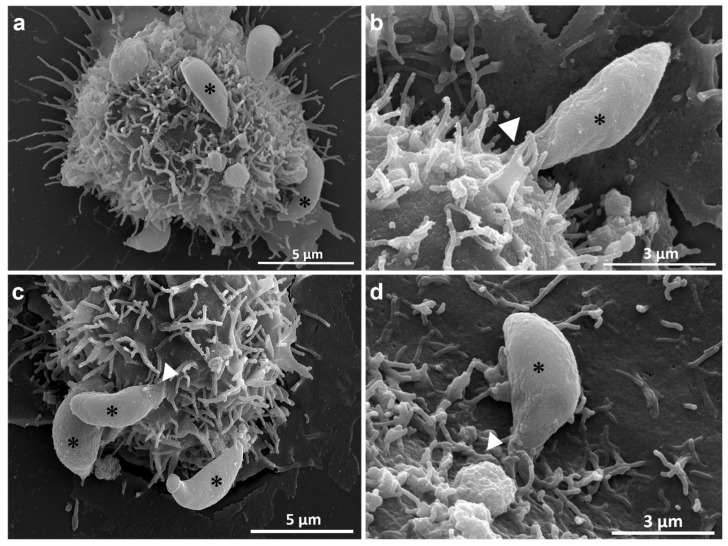
Surface of the untreated host cell in contact with the tachyzoites. (**a**). Several tachyzoites (*) are associated with the cell surface. (**b**). Cytoplasmic projections (arrowhead) of the host cell can be seen surrounding the posterior part of the parasite (*). (**c**). Host cell membrane (arrowhead) surrounding the conoid region of the tachyzoite (*) during the interaction. (**d**). 80 nm thick projections (arrowhead) surrounding the apical pole of the tachyzoite (*).

Scanning electron microscopy images of tachyzoites allowed to interact for 15 min with control cells showed images that confirm previous studies on the parasite–host cell interaction. Figure 2 shows images where parasites attached to the host cell surface are evident, with the presence of surface projections in contact with the tachyzoites. Short TNTs were seen in association with the tachyzoites. Several TNTs were observed throughout the interaction, possibly initiating the sequential process of recognition and adhesion to the host cell surface, with subsequent internalization.

**Figure 3:**
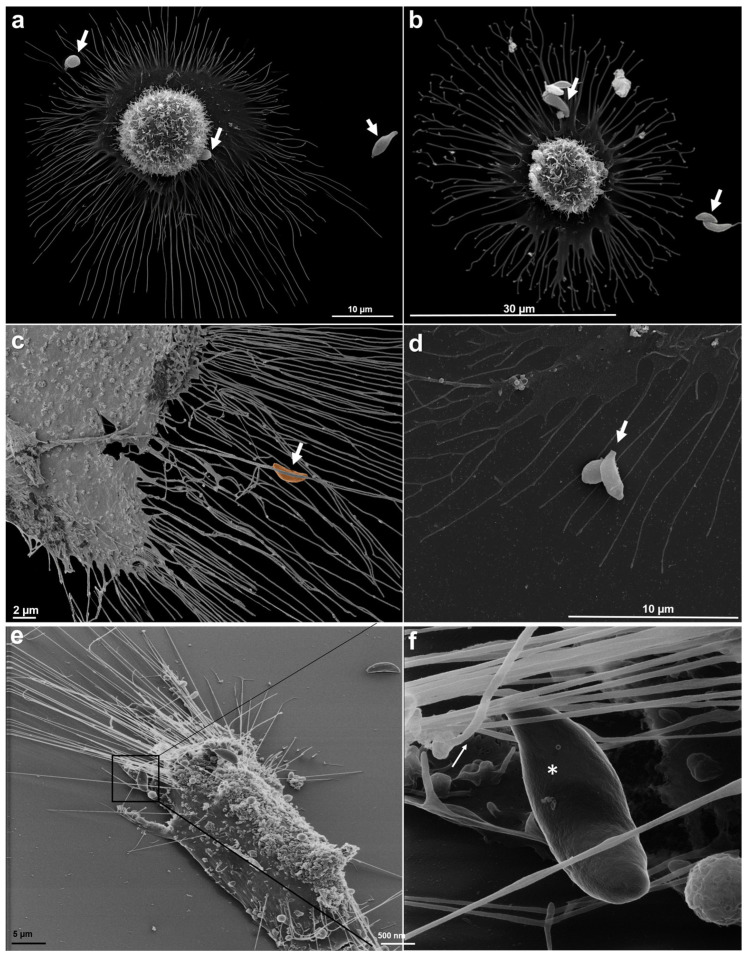
TNTs from the host cell pretreated with sucrose for 30 min and the tachyzoite during recognition. (**a**). LLC MK^2^ cell exhibiting TNTs up to 22 µm in length. (**b**). Tachyzoites dispersed among the TNT projections from the host cell. (**c**). Numerous TNTs projected from the host cell and tachyzoites during recognition. (**d**). Tachyzoite in contact with the apical pole with the TNT. (**e**). Overview of the host cell displaying numerous tunneling nanotubes and surrounding tachyzoites. (**f**). Higher magnification of a selected region from panel (**e**), showing a tachyzoite (*) closely interacting with host cell projections. Arrows: tachyzoites interacting with TNTs.

When parasites were allowed to interact with sucrose-treated cells, multiple TNTs can be observed projecting from the host cell, with tachyzoites positioned in proximity. This spatial organization suggests a potential role for these structures in attracting or guiding parasites. Higher magnification, as shown in Figure 3, reinforces this hypothesis by showing a tachyzoite in close contact with the projections, appear to interact with them in a directed manner. This pattern may indicate that TNTs function not only as structural elements of the host cell but also potentially as mediators of intercellular communication or as guiding structures that facilitate parasite approach and cellular recognition.

Scanning microscopy was used to analyze the projections of the TNTs in the various experimental conditions. In the presence of the tachyzoite, the previously treated cells showed TNTs that reached a length of up to 24 µm, and the parasites remained in contact with these projections (Figure 3).

**Figure 4:**
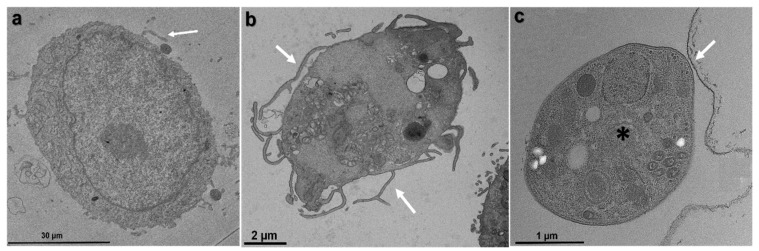
Comparison of cell morphology between control and treated cells, as well as the interaction of tachyzoites with TNTs. (**a**). Cell of the LLC MK^2^ line without any treatment. The arrow indicates a site of assembly of a TNT. (**b**). Cell of the LLC MK^2^ line treated with sucrose for 30 min with several TNTs (arrow). (**c**). Tachyzoite (*) in close contact with a TNT (arrow).

Transmission electron microscopy reveals differences between the TNTs of untreated cells compared to the treated ones, and that *T. gondii* tachyzoites interact closely with these projections, suggesting that TNTs may guide or facilitate the parasite’s entry into the host cell, possibly acting as pathways for communication or cellular recognition (Figure 4).

### 3.2. Quantitative Analysis of TNTs

**Figure 5:**
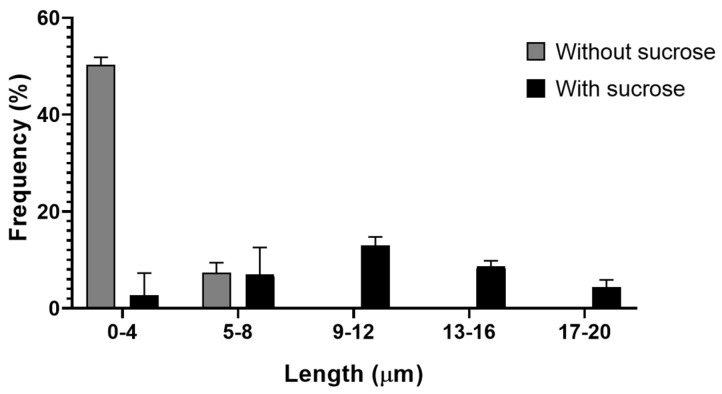
Frequency of length in micrometers of LLC MK^2^ cells. In gray, cells without sucrose treatment, and in black, cells treated for 30 min with sucrose.

We examined at least 150 SEM cell images to measure the length of the TNTs to determine their frequency distribution as shown in Figure 5. Data are the average of three independent experiments, each performed in triplicate (*** *p* < 0.001).

We observed that in the control cells, most of the TNTs presented lengths ranging from 0.9 to 5 µm, while in the cells previously treated with sucrose, TNTs reached a length of 20 µm.

**Figure 6:**
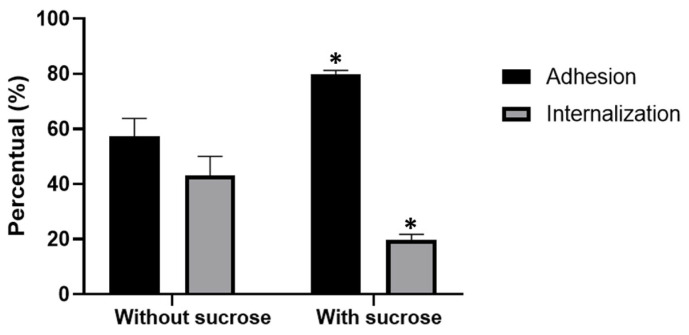
Quantification of *T. gondii* interaction with LLC MK^2^ cells. The interaction was performed under two conditions: (i) initially at 4 °C for 30 min and then maintained at 37 °C for an additional 20 min before adhesion (synchronized condition); or (ii) parasites interacted with host cells that had been pretreated with sucrose for 30 min prior to interaction. Adhesion and internalization indices were obtained as described in Materials and Methods. Data were obtained from three independent experiments. Student’s *t*-test was used to compare adhesion and internalization at the entry site of *T. gondii* into host cells. Values were considered statistically significant when * *p* ≤ 0.01 (*).

Similar experiments were conducted using control cells and cells treated with sucrose, incubated with *T. gondii* tachyzoites. We observed that parasite adhesion was significantly higher in cells treated with sucrose compared to control cells. On the other hand, the internalization index in control cells was approximately twice as high as in treated cells, reaching values similar to their respective adhesion indices. Adhesion and internalization indices were calculated based on the absolute number of events per 100 host cells; these values were subsequently normalized and expressed as a percentage for comparative purposes. The data underlying these absolute counts are available in Figure 6.

**Figure 7:**
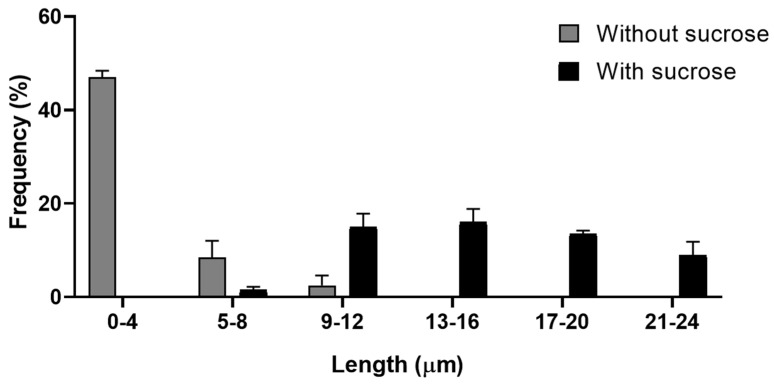
Frequency distribution of TNT lengths (in micrometers) in LLC-MK^2^ cells. In blue, cells without sucrose treatment and with interaction with the *T. gondii* tachyzoite; in black, TNTs of the cells treated for 30 min with sucrose and with interaction with the tachyzoite.

In comparison of both assays previously treated with sucrose, without the presence of the tachyzoite and in the presence of the parasite, there were TNTs that reached a size of up to 24 µm (Figure 7), under the conditions previously reported.

We made a quantitative analysis of the association of tachyzoites to the sucrose-induced TNTs. Our observations showed that 54% of the tachyzoites showed physical contact with the TNTs.

## 4. Discussion

It has been assumed that the surface of host cells plays an important role in the interaction with parasitic protozoa, especially with those that require cell invasion by the parasite. In the case of tachyzoites of *T. gondii*, it has been shown that they penetrate the host cell via endocytic processes induced, in part, by the secretion of parasite molecules that may activate almost all types of endocytosis. In a previous study, we showed that during parasite–host cell interaction, tachyzoites of *T. gondii* may attach to thin surface tubular structures known as TNTs [5]. Our present morphological observations add new information on this topic using an experimental approach where assembly of TNTs in the host cell surface was induced by incubation in the presence of 0.45 M sucrose, a treatment that promotes reversible hypertonic pressure, interfering with the mechanosensitive ion channels, inducing changes in the cytoskeleton, and interfering with clathrin-mediated endocytosis [11,13,14]. We further characterized this experimental system by showing that it can be reversed by washing the cells and incubating them in normal culture medium. During the experiments, cell viability remained around 94%.

Here, we confirmed that sucrose promoted TNT induction. After removal of this medium, subsequent washing, and incubation with medium supplemented with fetal bovine serum, there was a reduction in TNT elongation, as well as a 94% rate of viability of these cells. These results revealed that sucrose treatment was not detrimental to cell viability, and the cell projections were reversible, with no other ultrastructural changes observed. As in all experimental systems using different compounds, we always must consider the possibility of additional effects not yet characterized.

There are several terms used to describe membrane protrusions involved in cell–cell contact. Two types of membrane protrusions that may be confused are cytonemes and TNTs. The main difference between them is that cytonemes are extremely thin, and their interactions with other cells end when their membranes come into proximity [7]. In contrast, TNTs can undergo vesicular trafficking and, upon contacting another cell, fuse their membranes, thereby connecting the cytoplasm of both cells [15]. However, various organizations of TNT-like structures can be observed, in which membrane fusion and cytoplasmic connection may not occur.

Interaction between TNT-like structures and cytonemes of parasites with host cells have been previously reported [15,16]. It has been shown that *Leishmania donovani* can slide between the TNT-like structures of a macrophage and between B cells, thus indicating that during in vivo infection with *L. donovani*, with transmission of parasites from infected macrophages to B cells and their dissemination among B cells that may lead to activation of polyclonal B cells [16]. On the other hand, there are several reports dealing with assembly of TNT-like structures in parasitic protozoa such as *Trichomonas vaginalis*, *Tritrichomonas foetus*, *Giardia intestinalis*, and *Trypanosoma brucei* [15,17,18]. In the case of *T. vaginalis* it was shown that they protrude from both the surface of the protozoan and the flagella, especially on the attached parasites, and that they connected the parasites [15]. In the case of *Giardia duodenalis*, images showed the formation and extension of TNT-like structures all over the parasite surface both in vitro and in vivo [17].

The biogenesis of TNTs is not yet fully understood and likely depends on the cell type and microenvironment, including the sharing of intracellular material and the capture of microvesicles [7,19]. Previous studies described the emergence of TNTs a few hours after the start of cultivation, with TNTs remaining stable in semiconfluent cultures under acidic pH and hyperglycemic conditions, thereby favoring their formation [12]. In the present work, we used a medium supplemented with sucrose, which allowed us to morphologically characterize the TNT projections that interfere with the adhesion and internalization of *T. gondii* tachyzoites in host cells. Our present observations show that while the induction of TNTs significantly increased the adhesion of the parasites to the host cell surface, the process of invasion was reduced. Indeed, scanning electron microscopy revealed that 54% of the tachyzoites observed by SEM were in physical contact with the TNTs. It is important to point out that previous studies showed that incubation in the presence of sucrose interferes with clathrin-mediated internalization of *Toxoplasma gondii* [13] and *Trypanosoma cruzi* [11] by the host cells. Based on the observed decrease in parasite internalization and the consistent association with TNTs, our data suggest that TNTs may act as physical barriers, potentially limiting parasite access to the central domains of the host cell body where entry occurs. The data also suggests a potential association between TNT abundance and changes in parasite adhesion under sucrose-treated conditions. At present we cannot exclude the possibility that enhancement of parasite adhesion and partial inhibition of parasite internalization is solely due to TNTs. It is important to consider the fact that sucrose treatment also interferes with endocytic activity.

## Data Availability

The original contributions presented in this study are included in the article. Further inquiries can be directed to the corresponding authors.

## Abbreviations

The following abbreviations are used in this manuscript:

Abbreviation	Meaning
TNT	Tunneling Nanotube(s)
*T. gondii*	*Toxoplasma gondii*
SEM	Scanning Electron Microscopy
TEM	Transmission Electron Microscopy
LLC-MK^2^	Rhesus monkey kidney epithelial cell line
RPMI	Roswell Park Memorial Institute medium
PBS	Phosphate-Buffered Saline
pH	Hydrogen Ion Concentration
FCS	Fetal Calf Serum
MTS/PMS	MTS: 3-(4,5-dimethylthiazol-2-yl)-5-(3-carboxymethoxyphenyl)-2-(4-sulfophenyl)-2H-tetrazolium/PMS: Phenazine Methosulfate
BSA	Bovine Serum Albumin
DAPI	4′,6-diamidino-2-phenylindole
OsO_4_	Osmium Tetroxide
nm	Nanometer
μm	Micrometers
